# Preoperative Strategies for Locally Advanced Colon Cancer

**DOI:** 10.1007/s11864-024-01184-6

**Published:** 2024-02-13

**Authors:** Kanika G. Nair, Suneel D. Kamath, Nivan Chowattukunnel, Smitha S. Krishnamurthi

**Affiliations:** Department of Hematology and Oncology, Cleveland Clinic Taussig Cancer Center, 9500 Euclid Avenue, Cleveland, OH 44195 USA

**Keywords:** Colon cancer, Neoadjuvant, Preoperative, Mismatch repair, Microsatellite instability, Microsatellite stable

## Abstract

Neoadjuvant chemotherapy is safe for patients with locally advanced colon cancer (LACC). The FOxTROT trial demonstrated a reduction in residual and recurrent cancer at 2 years with neoadjuvant chemotherapy for patients with cT3-4 LACC. Preoperative chemotherapy should be avoided, if possible, for patients with dMMR LACC, as over 50% of dMMR cancers have no pathologic response. Early universal testing of MMR status is critical to selecting the appropriate neoadjuvant therapy. Concerns about CT staging of LACC have limited uptake of neoadjuvant chemotherapy, as approximately 25% of patients with cT3-T4 cancer on CT have low-risk stage II disease. Development of CT criteria for malignant nodes should reduce the risk of over-staging. A multidisciplinary approach is needed to identify patients for neoadjuvant therapy. Neoadjuvant immunotherapy is safe and results in dramatic pathologic responses in patients with dMMR LACC. Longer follow-up is needed to determine if the exceptionally high pathologic response rates observed will translate into long-term remission. Remarkably, neoadjuvant immunotherapy has been found to cause major pathologic responses in a subset of patients with pMMR LACC, indicating the potential to cure more patients with this common cancer. Patients with cT4 LACC, whether stage II or III, have a substantial risk of recurrence despite adjuvant fluoropyrimidine plus oxaliplatin chemotherapy. We recommend neoadjuvant systemic therapy for all patients with cT4b LACC (dMMR and pMMR). Features of T4b disease are routinely reported by radiology. We use three cycles of FOLFOX chemotherapy for patients with cT4b pMMR LACC, due to the high rate of compliance and improvement in residual and recurrent disease. Patients with cT4b dMMR LACC should receive neoadjuvant immunotherapy, if there are no contraindications. Clinical trials of neoadjuvant therapy for LACC are of great interest and should provide training for radiologists to identify eligible patients. Results are anticipated from multiple ongoing trials of neoadjuvant chemotherapy, immunotherapy, and targeted therapy for pMMR LACC and immunotherapy for dMMR LACC.

## Introduction

Preoperative strategies for locally advanced colon cancer (LACC), defined as clinical stage II and III disease, include treatment of unresectable and resectable non-metastatic disease. Patients with unresectable LACC should be treated with regimens that maximize the tumor response rate to enable resection while maintaining patient safety and may also include localized radiation therapy [1]. Preoperative therapy for patients with resectable disease is predominantly neoadjuvant systemic therapy, which is the focus of this review, and could be chemotherapy, immunotherapy, or targeted therapies.

Potential advantages of neoadjuvant systemic therapy include improved disease-free survival (DFS) due to an increase in R0 resection rate and prevention of metastases through early initiation of systemic therapy. Patients with T4 and node-positive tumors have a 3-year DFS of only 60%, and those with T4N0 disease have a 5-year DFS of only 74%, despite adjuvant fluoropyrimidine and oxaliplatin chemotherapy and may benefit most from a neoadjuvant approach [2, 3]. Neoadjuvant therapy also allows an early assessment of the anti-tumor activity of systemic therapy based on pathologic tumor regression. Immunotherapy in the neoadjuvant setting is of particular interest as it results in high rates of pathologic response in multiple cancers and, combined with adjuvant immunotherapy, improves event-free survival for patients with melanoma, compared to adjuvant immunotherapy alone [4–7]. Increased diversity of activated T cells, attributed to the presence of the primary tumor, may be responsible for this effect [8]. Challenges with neoadjuvant systemic therapy are identification of appropriate patients with CT staging and changing the established practice of upfront surgery for resectable LACC.

Several randomized controlled trials of perioperative vs adjuvant-only chemotherapy for LACC have reported results recently [9•, 10, 11]. Neoadjuvant immunotherapy is of great interest for patients with deficient mismatch repair (dMMR) LACC given the remarkable pathologic and clinical responses reported in trials of dMMR LACC and rectal cancer [12••, 13–15]. Most patients with LACC have proficient mismatch repair (pMMR) disease, and there is great interest in extending the potentially curative benefits of immunotherapy to this large population in the neoadjuvant setting [12••, 16, 17].

## Neoadjuvant therapy for pMMR LACC

Multiple randomized trials have demonstrated the safety of perioperative chemotherapy for pMMR LACC, but they differ in their approach and efficacy. Recent small studies have reported pathologic responses to preoperative immunotherapy for patients with pMMR colon cancer.

### Neoadjuvant chemotherapy for pMMR LACC

The largest study to date of perioperative therapy in LACC is the FOxTROT trial, which randomized 1053 patients with clinical (c) T3–4 cancer in a 2:1 manner to three cycles of FOLFOX prior to surgery versus standard of care with upfront surgery [9•]. Adjuvant therapy was planned for all patients, regardless of pathological stage. A second randomization of neoadjuvant chemotherapy with or without panitumumab for patients with wild-type KRAS tumors was also performed. There was no benefit of panitumumab on outcomes, so results from the two arms of this randomization were merged in the initial analysis. A subsequent analysis has found a significant overall survival (OS) benefit with 6 weeks of panitumumab plus FOLFOX versus FOLFOX alone in the subgroup of patients with high expression of epiregulin and amphiregulin [18].

FoxTROT demonstrated a 4.6% absolute reduction with preoperative therapy in the primary outcome of residual or recurrent disease within 2 years (16.9% vs 21.5%; rate ratio (RR) = 0.72 (95% CI of 0.54–0.98 and *p* = 0.037)). Similar proportional reductions in colon cancer-specific mortality (RR = 0.74 (95% CI 0.52–1.05, *p* = 0.095)) and all-cause mortality were observed (RR=0.76 (95% CI 0.55–1.06, *p*= 0.104)), though not statistically significant. Patients receiving preoperative therapy were more likely to have an R0 resection (94% vs 89%, *p* < 0.001).

A major finding from this study was the strong correlation between the pathologic tumor regression score (Dworak score) and risk of recurrence. The 3.5% of patients with a complete pathological response had no recurrence in 5 years in contrast to the 35% of patients with no tumor regression who had a 29% risk of recurrence over 5 years. Proficient MMR cancers were statistically more likely to have moderate or greater tumor regression than dMMR cancers (23% vs 7%, *p*<0.001). Among dMMR cancers, 70.4% had no regression following preoperative therapy, compared to 27.3% of pMMR/MMR-unknown cancers. Three cycles of neoadjuvant FOLFOX resulted in significant down-staging of tumor and nodes (*p* <0.0001) compared to the upfront surgery arm, e.g., pT4 of 20.7% versus 30.5% and N2 disease of 15.1% vs 25.9%.

Treatment delivery in FOxTROT was high with 90% of patients randomized to preoperative chemotherapy receiving all 3 cycles. Over 99% of patients in both arms proceeded to surgery. FOxTROT established that preoperative chemotherapy was safe and led to fewer surgical complications. The risk of anastomotic leak was numerically lower with preoperative chemotherapy (4.7% vs 7.4%, *p* = 0.07) as was the rate of complications requiring further surgery (4.3% vs 7.1%, *p* = 0.05) and complications prolonging hospital stay (11.6% vs 14.3%, *p* =0.22). Of note, deep venous thrombosis or pulmonary embolism was more common in patients receiving preoperative therapy (2.5% vs 0.6%), likely due to the thrombogenic effects of chemotherapy.

One criticism of FOxTROT is that 24% of patients in the upfront surgery arm had pathologic low-risk disease (pT1-3N0) and did not meet standard criteria for adjuvant therapy. FOxTROT enrolled patients with cT4 tumors or cT3 tumors with at least 5 mm extension beyond the bowel wall, which was subsequently changed to 1 mm extension. While over 50% of the patients in the upfront surgery arm had node-positive disease, the risk of over-treatment of patients with low-risk stage II disease remains a concern with the cT3-T4 (N any) eligibility criterion.

The OPTICAL trial in China randomized 744 patients with T3 or T4 disease (*N* any) as assessed by CT to 3 months of neoadjuvant chemotherapy with FOLFOX or CAPOX followed by surgery and 3 months of adjuvant chemotherapy versus upfront surgery followed by optional adjuvant chemotherapy based on pathological stage at the investigator’s discretion [10]. Results have been presented but not yet published. With this staging criterion, 26% of patients in the upfront surgery arm had pathologic low-risk stage II disease, again raising the concern of potential over-treatment.

Similar to FOxTROT, 94% of patients in OPTICAL received at least 6 weeks of neoadjuvant treatment. Only 62% of patients, however, completed three months of neoadjuvant chemotherapy. Patient noncompliance was the most common reason for not completing treatment. There was no difference in the rate of laparoscopic surgery, R0 resections, or surgical complications between the two groups.

While there was an improvement in the primary endpoint of 3-year DFS, it was only 2% higher in the preoperative therapy group and not statistically significant (78.7% vs 76.6%; HR 0.83, 95% CI 0.60–1.15; *p*=0.138). However, preoperative therapy did significantly improve 3-year OS (94.9% vs 88.5%; HR 0.43, 95% CI 0.22–0.83; *p*=0.012). At 20 months, the survival curves separate, and this separation is maintained even at 5 years. Interestingly, DFS was statistically improved with preoperative therapy in females (84.2% vs 74.7%; HR 0.54, 95% CI 0.31–0.93; *p*=0.02).

In the preoperative therapy group, 7% of patients had a complete response, and more patients had stage pT0-2 (23% vs 6%) or pN0 (69% vs 54%). An absolute reduction of 10% in pT4 and 5% in pN2 disease was seen. Mismatch repair status was available for 80% of patients who received preoperative therapy and indicated 15% of patients had dMMR cancer. Among pMMR cancers, 16.8% had a complete or marked response. Consistent with FOxTROT, dMMR cancers appeared more resistant to chemotherapy with 51.2% having poor or no response, compared to 25.7% of pMMR cancers.

The Scandinavian NeoCol trial randomized 250 patients with T3 or T4 and N0-2 disease as assessed on CT scan to 3 cycles of CAPOX or FOLFOX prior to surgery versus standard of care with upfront surgery [11]. The data have been presented but not published. Adjuvant therapy was administered based on pathological stage for both groups.

There was no difference in 5-year DFS (85%) or OS (90%) between the groups (logrank *p*=0.95 for both) in NeoCol. Three percent of patients receiving preoperative therapy had a complete response. Tumor regression score was not reported, and MMR status was not available. Surprisingly, there was little reduction in pT4 disease with 3 cycles of neoadjuvant CAPOX compared to initial surgery (28% and 32%). Node-negative disease was more common in patients receiving preoperative therapy (59% vs 48%), and lymphovascular invasion was less common (25% vs 39%). The R0 resection rate was numerically higher in the neoadjuvant arm (93% vs 90%).

Neoadjuvant chemotherapy was safe in NeoCol. More patients who received neoadjuvant therapy underwent laparoscopic resection (75% vs 68%). Ileus (3% vs 8%) and anastomotic leaks (3% vs 8%) were less common in patients receiving preoperative therapy, while perioperative blood transfusion was more common in patients receiving preoperative therapy (7% vs 4%).

All patients in NeoCol received CAPOX, and the mean number of preoperative CAPOX cycles received was 2.7. Fewer patients who received neoadjuvant therapy met criteria for adjuvant therapy (59% vs 73%) and, on average, received 1 cycle less of CAPOX. Toxicity from chemotherapy, specifically the rates of sensory and motor neuropathy, was lower for patients receiving neoadjuvant therapy during treatment and during follow-up (9% vs 13% and 3% vs 8%, respectively), likely due to the break in chemotherapy for surgery and reduced number of cycles.

### Summary

These three randomized controlled trials of perioperative vs only adjuvant chemotherapy for pMMR LACC have all demonstrated the safety of preoperative chemotherapy. Compliance is excellent for 6 weeks of preoperative therapy but not 3 months. Preoperative chemotherapy should be avoided if possible for patients with dMMR LACC, as over 50% of dMMR cancers had no pathologic response. Efficacy has varied between the three trials, possibly due to differences in receipt of adjuvant chemotherapy. FOxTROT demonstrated improvement in residual or recurrent disease and OPTICAL found an OS benefit of preoperative chemotherapy, whereas NeoCol found no difference in 5-year DFS or OS. FOxTROT and OPTICAL, but not NeoCol, demonstrated down-staging of cancer with neoadjuvant chemotherapy.

One concern that has limited uptake of preoperative therapy for LACC in the USA is the accuracy of CT staging. Both FOxTROT and OPTICAL found that approximately 25% of the cT3-T4 tumors in the upfront surgery arms were actually low-risk stage II cancers. Identification of malignant lymph nodes on CT is evolving. Recent studies have found that CT has higher specificity for malignant nodes in pMMR than dMMR LACC [19, 20]. Criteria to identify malignant lymph nodes in LACC are under development [20]. A multidisciplinary approach is needed for neoadjuvant therapy, as most patients in the USA currently undergo surgery without seeing a medical oncologist. Radiologists typically report colon tumor invasion or adherence to adjacent organs or structures, i.e., cT4b disease, which facilitates neoadjuvant therapy for this tumor stage. Our approach is to treat patients with cT4b pMMR LACC with a short course of preoperative chemotherapy, as in the FOxTROT trial.

### Neoadjuvant immunotherapy for pMMR colon cancer

While immunotherapy for pMMR tumors in the metastatic setting has shown poor response rates, the NICHE-1 study suggests a possible role for neoadjuvant immunotherapy in patients with locally advanced, potentially resectable pMMR tumors [12••, 21•]. In this study, 31 patients with pMMR tumors received neoadjuvant ipilimumab on day 1 + nivolumab on day 1 and day 15. Patients were randomly assigned to take celecoxib 200 mg daily from day 1 to the day before surgery. The rationale for celecoxib use was that inhibition of cyclooxygenase and subsequent prostaglandin E2 production would reduce tumor-promoting inflammation [22]. Celecoxib in a preclinical model of colorectal cancer resulted in synergy with anti-PD-1 therapy [22]. The primary objective of safety and feasibility was met, with all patients undergoing surgery within the predefined 6 weeks after study inclusion. Only 13% of patients experienced a grade 3–4 treatment-related toxicity, all of which resolved with medical management. The secondary objectives were pathologic response rate and DFS. The results were impressive with a 29% response rate. There were 4 complete pathologic responses (pCR), 3 additional major pathologic responses (MPR) with < 10% viable tumor remaining, and 2 partial responses with ≤ 50% viable tumor remaining. The remaining 22 patients were non-responders (> 50% viable tumor remaining). There was no indication of a celecoxib effect on response. Adjuvant chemotherapy was given to 8 patients with pMMR cancer, who were all non-responders. After a median of 28 months of follow-up, only 2 of these patients had a recurrence, both of whom were non-responders to immunotherapy (2/31 pMMR patients).

Considering that few patients with pMMR metastatic colorectal cancer respond to even months of checkpoint inhibitor therapy, the efficacy of immunotherapy in NICHE-1 was unprecedented and suggests that the immune environment of the primary tumor in early-stage colorectal cancer is more sensitive to immunotherapy than that of primary tumors and metastases in the metastatic setting. Analysis of the pathology samples comparing pMMR responders and non-responders showed only one biomarker predictive of response rate: the presence of T cells with co-expression of CD8 and PD-1. Much about the mechanism of immune activation of these tumors is still unknown, but CD8+PD-1+ T cell infiltration could become a predictive biomarker for response rates to immunotherapy if validated in larger trials.

The NEST-1 trial (NCT05571293) is a single-arm study evaluating neoadjuvant combination immunotherapy with botensilimab, an enhanced anti-CTLA-4 agent, plus balstilimab, an anti-PD-1 agent for patients with stage 1–3 colon adenocarcinoma (pMMR). Like NICHE, this trial administers one dose of the anti-CTLA-4 antibody and two doses of the anti-PD-1 antibody before surgery. A report of dramatic pathologic response in two patients with pMMR cancer on this trial has been reported [16]. Of note was the finding of a massive immune infiltrate in these cancers resulting in residual viable tumor only near the luminal surface of the colon.

A signal of efficacy of neoadjuvant single-agent anti-PD-1 therapy for pMMR colon cancer was observed in the NICOLE trial [17]. Twenty-two patients with cT3/4 colon cancer were treated with two 240 mg doses of nivolumab prior to surgery. Two of 18 patients with pMMR colon cancer had a MPR with one pCR.

### Summary

These three small trials provide an encouraging signal of activity of immunotherapy in the neoadjuvant setting for pMMR LACC. Larger studies and longer follow-up are needed to determine the long-term safety and efficacy. Future studies will aim to determine the optimal combination and duration of neoadjuvant immunotherapy for these tumors. The neoadjuvant setting is also ideal to assess the efficacy of targeted therapies for early-stage disease such as LACC with BRAF V600E mutations or HER2 amplifications. Table 1 lists ongoing trials of neoadjuvant immunotherapy or targeted therapy for pMMR colon cancer.

**Table 1:** Ongoing trials of neoadjuvant immunotherapy or targeted therapy for pMMR colon cancer

Trial identifier	Patient population	Regimen	Primary endpoint
Immunotherapy only			
NCT03026140 NICHE	Non-metastatic	Nivolumab + BMS-986253 (anti-IL8) OR Nivolumab + relatlimab	AE and DFS until 5 years
NCT05571293 NEST	Clinical stage I–III	Botensilimab + balstilimab	Pathologic overall response rate, AE, SAE, delays in surgery
Immunotherapy + chemotherapy			
NCT05914389	cT3-4 or node-positive	Envafolimab + CAPOX × 4 vs Envafolimab + CAPOX × 4 + Lactobacillus vs CAPOX × 4	TRG 0 or 1 (AJCC CAP)
NCT04625803	cT3 ≥ 5mm invasion or cT4	Camrelizumab + FOLFOX + apatinib	TRG at 3 months
NCT03985891	Locally advanced with MDT indication for neoadjuvant therapy	FOLFOX +/− toripalimab	pCR radiographic CR Immunotherapy overall RR
NCT03984578	cT4 or cN2	CAPOX + pembrolizumab	TRG; immune gene signature and immune cell infiltrate
Immunotherapy or targeted therapy			
NCT05845450 UNICORN	cT3-4	HER2 overexpression/amplification: trastuzumab deruxtecan POLE/D1 mutation with ultra-mutated status (>100 Mut/megabase): durvalumab RAS and BRAF wild-type, PRESSING negative status and left-sided primary tumor: panitumumab Absence of HER2 overexpression/amplification and absence of POLE/D1 mutation: botensilimab or botensilimab + balstilimab KRAS G12C mutation: sotorasib + panitumumab	MPR
Targeted therapy			
NCT00647530 FOxTROT 4	BRAF V600E	Encorafenib + cetuximab vs fluoropyrimidine/oxaliplatin	TRG
NCT05510895	cT3-4 and/or N+ (> 1 cm) BRAFV600E	Encorafenib + binimetinib + cetuximab	TRG
NCT05706779	cT3-4 BRAF V600E MMR not specified	Encorafenib + cetuximab	TRG

*MPR* major pathologic response (<10% viable tumor remaining); *AE* adverse events; *SAE* serious adverse events; *MDT* multidisciplinary team; *pCR* pathologic complete response; *CR* complete response; *RR* response rate; *TRG* tumor regression grade; *PRESSING* HER2/MET amplification, ALK/ROS1/NTRKs/RET fusions, mutations in HER2/PIK3CAex.20/PTEN/AKT1 and RAS, and microsatellite-instability high status

## Neoadjuvant therapy for dMMR LACC

Neoadjuvant therapy in LACC is overall a novel paradigm with some of the most exciting data coming from the dMMR population. It is well established that dMMR colorectal cancers express significantly greater numbers of neoantigens, rendering them more sensitive to immune checkpoint inhibition [23, 24]. While dMMR is rare in metastatic colon cancer, present in approximately 5% of patients, it is more common in earlier stage disease, comprising 15–18% of stage II and 11% of stage III colon cancers [25, 26].

Compared to pMMR colon cancer, dMMR tumors have a more favorable prognosis but are less sensitive to chemotherapy [27]. As a result, adjuvant chemotherapy is not recommended for most patients with stage II dMMR colon cancer. As previously discussed, both the FOxTROT and OPTICAL trials found dMMR LACC to be relatively resistant to preoperative chemotherapy [9•, 10].

Neoadjuvant immunotherapy has shown exceptional activity in several, single-arm clinical trials. NICHE-1 was the first prospective trial to investigate neoadjuvant immunotherapy in early-stage colon cancer [12••]. The treatment consisted of two cycles of nivolumab (3 mg/kg) given every 2 weeks and one dose of ipilimumab (1 mg/kg) given on the first day of study treatment followed by surgery within 6 weeks of study enrollment. The trial included 63 patients with resectable, stage I–III colon cancer, 32 with dMMR tumors. In the dMMR group, 47% of patients had T4 tumors, and 78% had clinically positive lymph nodes. A MPR occurred in 97% of patients, including 69% with pCR both in the primary tumor bed and lymph nodes. Only one patient (3%) had a partial response, and there were no non-responders in the dMMR population. After a median follow-up of 32 months, recurrence-free survival (RFS) was 100% [21•].

The NICHE-2 trial expanded on NICHE-1 by evaluating the same nivolumab and ipilimumab regimen in a larger cohort of 112 patients with resectable, non-metastatic dMMR colon cancer [28•]. Thirty-five (31%) patients had Lynch syndrome. The majority of patients had high-risk disease; 63% had T4 tumors, 62% had N2 disease, and 48% had both T4 tumors and N2 disease. Despite these high-risk features, MPR was observed in 95% of patients, including 67% with pCR of the primary tumor. All patients underwent surgery, and 100% had R0 resections, indicating that the short delay in surgery did not negatively impact surgical outcomes. After a median follow-up of 13.1 months, RFS was 100%. The pCR rate was numerically higher in patients with Lynch syndrome compared to those with sporadic dMMR tumors (78% vs. 58%), but this did not reach statistical significance (*p* = 0.056). Neoadjuvant immunotherapy was extremely well-tolerated. Sixty-one percent of patients experienced any grade immune-related adverse event (irAE), but only 4% were grade 3 or higher. Five grade 3 or 4 events were observed in 4 patients: asymptomatic amylase and lipase increases which resolved without intervention, rash and hepatitis treated and resolved with prednisone, and myositis treated with prednisone and mycophenylate with complete resolution. There were no treatment-related deaths, and only one patient (2%) experienced an irAE requiring a delay in surgery for more than 2 weeks.

Another single-arm, phase II trial examined neoadjuvant immunotherapy with pembrolizumab for six months in dMMR solid tumors [14]. Patients were also considered for non-operative management and could receive pembrolizumab for an additional 1 year. The study included 35 patients, 19 with colon cancer. Of the 12 patients who underwent surgery, 10 (83%) had a pCR. Of the seven patients who underwent non-operative management, six continued to receive pembrolizumab at time of data cutoff, and one had progression of disease after two cycles of pembrolizumab and died of disease progression while receiving chemotherapy.

The PICC trial also studied neoadjuvant immune checkpoint inhibition and added cyclooxygenase-2 (COX-2) inhibition to see if this could augment pathologic response [15]. As mentioned previously, COX-2 and prostaglandin E2 (PGE2) overexpression has been shown to upregulate tumor-promoting cytokines and growth factors, and COX-2 inhibition with celecoxib can augment tumor regression with immune checkpoint blockade in colorectal cancer xenograft models [22, 29]. This randomized, non-comparative phase II trial conducted in China included 34 patients, 17 who were treated with the anti-PD-1 drug toripalimab alone for 3 months and 17 who were treated with toripalimab and celecoxib for 3 months. All 34 patients successfully underwent surgery and achieved R0 resections. In the toripalimab monotherapy arm, 65% achieve a pCR, while 88% in the combination arm achieved a pCR. Of note, the combination arm was a lower risk population due to a higher proportion of T3 or N0 disease compared to the monotherapy arm, which may have contributed to the higher pCR rate observed in the combination arm. After a median follow-up of 14.9 months, DFS and OS were both 100% in both treatment arms.

The NICHE-3 trial is evaluating treatment with two doses of neoadjuvant nivolumab and relatlimab, an anti-LAG3 antibody, in patients with resectable, dMMR LACC [30]. An impressive pCR rate of 79% and a MPR rate of 89% were found in the first 18 patients. All patients underwent surgery without delay. Treatment was well-tolerated with 74% of patients experiencing grade 1–2 irAEs and only 1 patient having a grade 3 irAE (hyperthyroidism). Four patients required supplementation for endocrine toxicity: 1 for hypothyroidism and 3 for hypophysitis with secondary adrenal insufficiency. There were no grade 4–5 irAEs. Accrual is ongoing.

### Summary

Overall, the data for neoadjuvant immunotherapy in non-metastatic colon cancer are extremely compelling. The only conflicting data come from the NICOLE trial, which investigated nivolumab in the neoadjuvant setting [17]. Of the 22 patients included in the trial, only 3 had dMMR tumors. No major pathologic responses were seen in these 3 patients, though complete response data were not reported. We treat patients with cT4b dMMR LACC with preoperative immunotherapy. We recommend ipilimumab and nivolumab according to the NICHE trial, in light of the safety profile and profound pathologic response observed with this short course of treatment. Single-agent anti-PD-1 therapy could also be used for a longer duration, e.g., if a non-operative approach is desired.

Several areas for future research remain in this exciting, evolving field. The optimal duration of neoadjuvant and adjuvant immunotherapy remains to be determined. Single agents will be less toxic, but combination therapy may be more efficacious. There are many trials underway of neoadjuvant single-agent anti-PD1 therapy for dMMR LACC. Table 2 lists ongoing trials of combination neoadjuvant therapy for dMMR colon cancer. Innovations in imaging techniques could improve radiographic staging at baseline to optimize patient selection for neoadjuvant therapy and also to identify which patients can safely pursue organ preservation. Circulating tumor DNA dynamics could also guide immunotherapy response assessment, treatment duration, and patient selection for organ preservation. Long-term follow-up is needed to determine if the exceptionally high pathologic response rates observed will translate to long-term remission.

**Table 2:** Ongoing trials of combination neoadjuvant therapy for dMMR colon cancer

Trial identifier	Patient population	Neoadjuvant regimen	Primary endpoint
Combination immunotherapy			
NCT03026140 NICHE	cT4NanyM0 or cTanyN+M0	Nivolumab + relatlimab	AE until 100 days after last study drug; DFS up to 5 years
NCT05571293 NEST	Clinical stage I–III	Botensilimab + balstilimab	Pathologic overall RR, AE, SAE, delays in surgery
NCT05845450 UNICORN	cT3-T4	Botensilimab followed by a cohort of botensilimab + balstilimab	MPR
NCT05913570	cT4 or cN1-2 (N+ defined as > 1.0 cm)	Cadonilimab	pCR
NCT 5890742	cT4 or cN1-2	Sintilimab +/− IBI310	pCR
Immunotherapy + other agents			
NCT 3926338	cT3-4 or cN1-2 (N+ = ≥ 1.0 cm)	Toripalimab +/− celecoxib	3-year DFS; pCR
NCT4715633	cT3 with ≥ 5 mm extension or cT4	Camrelizumab + Apatinib	cCR or pCR up to 2 years
NCT 4988191	cT4 resectable	Toripalimab + bevacizumab + irinotecan	pCR
NCT06014372	cT3-4 or cN1	Envafolimab + CAPOX	pCR
NCT5841134	cT3-T4 N0 M0 or Tany N+M0	Tislelizumab + CAPOX	cCR or pCR up to 2 years

*AE* adverse event; *DFS* disease-free survival; *RR* response rate, *SAE* serious adverse event; *MPR* major pathologic response (<10% viable tumor remaining); *pCR* pathologic complete response; *cCR* clinical complete response
